# Effect of nocturnal hypoxemia on glycemic control among diabetic Saudi patients presenting with obstructive sleep apnea

**DOI:** 10.3389/fendo.2022.1020617

**Published:** 2023-01-18

**Authors:** Mahmoud I. Mahmoud, Raed K. Alotaibi, Rayyan Almusally, Hanaa Shafiek, Yasir Elamin, Ziad Alhaj, Waleed AlBaker, Alaeldin Elfaki, Hatem Qutub, Suha J. Albahrani, Fatima M. Alabdrabalnabi, Zahra A. Al Saeed, Reem Al Argan, Fatima Al.Rubaish, Yousef D. Alqurashi, Noor-Ahmed Jatoi, Abdullah H. Alharmaly, Zainab Z. Almubarak, Abir H. Al Said, Nada Albahrani

**Affiliations:** ^1^ Department of Internal Medicine, King Fahd Hospital of the University, Imam Abdulrahman Bin Faisal University, Khobar, Saudi Arabia; ^2^ Chest Diseases Department, Faculty of Medicine, Alexandria University, Alexandria, Egypt; ^3^ Family and Community Medicine Department, College of Medicine, King Fahd Hospital of the University, Imam Abdulrahman Bin Faisal University, Dammam, Saudi Arabia; ^4^ Department of Internal Medicine, College of Medicine, Imam Abdulrahman Bin Faisal University, King Fahd Hospital of the University, Khobar, Saudi Arabia; ^5^ United Lincolnshire Hospitals, NHS Trust, Lincoln, United Kingdom; ^6^ Family Medicine Department, College of Medicine, King Faisal University, Al-Ahsa, Saudi Arabia; ^7^ Internal Medicine Department, King Fahad Specialist Hospital, Dammam, Saudi Arabia; ^8^ Respiratory Care Department, College of Applied Medical Sciences, Imam Abdulrahman Bin Faisal University, Dammam, Saudi Arabia; ^9^ Pulmonary Department, Prince Sultan Military Medical City, Riyadh, Saudi Arabia; ^10^ Otolaryngology-Head and Neck Surgery Department, King Fahd Hospital of the Imam Abdulrahman Bin Faisal University, Khobar, Saudi Arabia

**Keywords:** glycosylated hemoglobin, apnea/hypopnea index, hypoxia, nocturnal hypoxemia, obstructive sleep apnea, T90

## Abstract

**Background:**

Obstructive sleep apnea (OSA) is a prevalent disease that is associated with an increased incidence of type II diabetes mellitus (DM) if left untreated. We aimed to determine the association between glycosylated hemoglobin (HbA1c) levels and both nocturnal hypoxemia and apnea-hypopnea index (AHI) among a Saudi patients with OSA.

**Methods:**

A cross-sectional study that enrolled 103 adult patients diagnosed with DM and confirmed to have OSA by full night attended polysomnography between 2018 and 2021. Those who presented with acute illness, chronic obstructive pulmonary disease (COPD)/restrictive lung diseases causing sleep-related hypoxemia, or no available HbA1c level within 6 months before polysomnography were excluded from the study. Univariate and multivariate linear regression analyses between HbA1c levels and parameters of interest were tested.

**Results:**

Sixty-seven (65%) of the studied population had uncontrolled DM (HbA1c ≥7%). In univariate regression analysis, there was a significant positive association between HbA1c, and sleep time spent with an oxygen saturation below 90% (T90), female gender, and body mass index (BMI) (p<0.05) but not AHI, or associated comorbidities (p>0.05). In the multivariate analysis, HbA1c was positively associated with increasing T90 (p<0.05), and ODI (p<0.05), but not with AHI (p>0.05).

**Conclusion:**

Nocturnal hypoxemia could be an important factor affecting glycemic control in patients with OSA suffering from DM irrespective of the severity of both diseases.

## 1 Introduction

Sleep-disordered breathing (SDB) is a prevalent condition (1) that is underdiagnosed and undertreated. Obstructive sleep apnea (OSA) is the most common form of SDB (2). Globally, it is estimated that around 425 million adults have moderate to severe OSA (1). The estimated prevalence of mild OSA in the Kingdom of Saudi Arabia (KSA) is around 24.4% while moderate to severe OSA is around 6.4% (1). Untreated OSA leads to increased health care utilization and costs (3, 4).

OSA is associated with higher mortality (5–7) whereas moderate to severe OSA was significantly associated with all-cause mortality (8). OSA is related to multiple comorbidities including hypothyroidism, systemic hypertension (HTN), heart failure (HF) with reduced ejection fraction, atrial fibrillation (AF), and coronary artery disease (CAD) (9). Further, OSA –especially if left untreated– is associated with a significantly increased incidence of type II diabetes mellitus (DM) (5) which could increase the incidence of stroke, CAD (5, 6), diabetic kidney disease (10, 11) and diabetic retinopathy (12) independent of several other risk factors (11).

We hypothesized that obstructive events could affect glycemic control in patients suffering from SDB, especially OSA. Therefore, we aimed to determine if there was an association between glycosylated hemoglobin (HbA1c) levels and nocturnal hypoxemia among Saudi diabetic patients presenting with OSA.

## 2 Methods

### 2.1 Study design, sample size needed and ethics

A cross-sectional, single-center study was conducted between November 2018 and December 2021 at the Sleep Laboratory of the Internal Medicine Department, King Fahd Hospital of the University, Imam Abdulrahman Bin Faisal University (IAU), Al-Khobar, Eastern Province, KSA, a tertiary referral hospital. To estimate the sample size needed we used Green’s rule of thumb (13) with the number of predictors as five and the sample needed was at least 90. The study protocol was approved by the institutional review board (IRB 2020-01-136) of IAU according to the Helsinki regulations.

### 2.2 Study population

The study included adult patients referred to the sleep clinic with symptoms suggestive of OSA. All patients who were adults (aged ≥ 18 years), had DM, and proved to have newly diagnosed OSA by polysomnography (PSG) were included in the current study. DM was defined as having a fasting plasma glucose of 126 mg/dl or more, an HbA1c of ≥6.5%, or a self-reported diagnosis of DM (14). Patients with a previous diagnosis of sleep-related hypoxemia due to pneumonectomy, bronchiectasis, interstitial lung diseases or chronic obstructive pulmonary disease (COPD), or presented with acute illness (such as acute heart failure or acute stroke), or on long-term oxygen therapy, or chronic opioid therapy, patients with acromegaly, patients with Down’s syndrome, patients with neuromuscular disorders, pregnant ladies, and patients with polycystic ovarian syndrome were excluded from the study (15). Further, individuals with no record of HbA1c level within 6 months before PSG were excluded from the study.

### 2.3 Data collection

Patients’ electronic records were reviewed after receiving the IRB approval. For each patient we collected the following data: age, gender, body mass index (BMI) calculated as weight in kilograms (kg) divided by the height in meters squared (m^2^), HbA1c levels, polysomnographic data, and information on comorbidities including DM, HTN, hypothyroidism, HF with a reduced ejection fraction, CAD, AF, and chronic kidney disease (CKD).

### 2.4 Polysomnography

All patients were subjected to nocturnal attended full polysomnography (PSG) (type I sleep study) (16) (SOMNOscreen TM Plus, SOMNOmedics GmbH, Germany). The PSG recording included electroencephalography (EEG), electrooculogram (EOG), electromyogram (EMG; chin and bilateral anterior tibialis), electrocardiogram (ECG), pulse oximetry, body position, airflow, and thoracic and abdominal movements using respiratory inductance plethysmography. Airflow was measured using both nasal thermistor and pressure transducer. Patients were monitored *via* camera throughout the study with video recording, and synchronized PSG video. The PSG studies were manually scored by one of the authors (MIM) using the American Academy of Sleep Medicine (AASM) scoring manual version 2.5 (17). Apnea hypopnea index (AHI) was calculated as the number of apneas and hypopneas per hour of sleep. The oxygen desaturation index (ODI) (number of 3% or more drops in the oxygen saturation per hour of sleep) as well as the percentage of the sleep time spent with oxygen saturation <90% (T90) were recorded. Those with an AHI > 5 events/hour with symptoms [symptoms were defined as presented in (17)] or those with an AHI > 15 regardless of symptoms were considered to have OSA (17).

### 2.5 Outcomes

The primary outcome of this study was to look for the association between diabetes control as assessed by HbA1c and severity of sleep apnea as assessed by AHI, ODI and T90.

### 2.6 Statistical analysis

We classified the population into controlled and uncontrolled DM based on HbA1c levels <7% and ≥7%, respectively. (14) and summarized the characteristics of continuous variables using the mean and standard deviation (SD). Categorical variables were summarized using frequency and percentage (%). Sleep parameters (T90, AHI, ODI) were converted using the natural log +1 due to a non-normal distribution and zero values (e.g., LnT90 = natural log (T90 +1)).

We used the variance test for comparison across continuous variables with a normal distribution and the Kruskal-Wallace rank-sum test for non-normal variables. For categorical variables, we used Fisher’s exact test. To test for normality, we used the Shapiro-Wilk test of normality. We conducted a univariate analysis using simple linear regression to identify the association between HbA1c level and PSG parameters for LnT90, LnAHI, and LnODI. A multivariate linear regression analysis was conducted using ordinary least squares (OLS) regression to control for several confounders in three models. In model 1 we controlled for age and gender. In model 2 we added BMI, and in model 3 (full model) we added history of HTN, CAD, CKD, stroke, HF, and AF. We present the summary finding of the three models in the main paper and a detailed version in the **Supplementary Material** that includes coefficients of all the variables. The corresponding 95% confidence intervals (95% CI) were reported. A p-value of <0.05 was considered significant. All analysis was conducted using R version 4.1.1 (2021-08-10).

## 3 Results

### 3.1 Population characteristics

**Figure 1:**
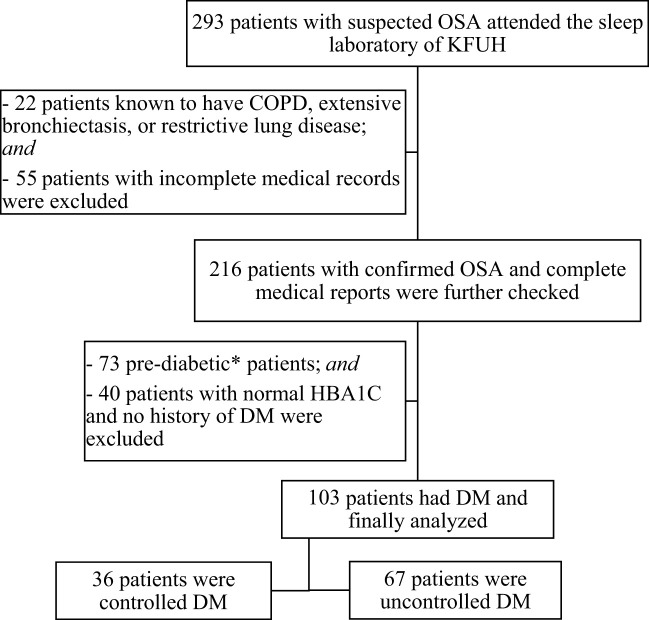
Patient flowchart.

A total of 293 patients with proven OSA were screened for eligibility criteria. One hundred and three patients (35%) were finally included in the study and further classified as controlled and uncontrolled DM (**Figure 1**). **Table 1** presents the baseline clinical and demographic characteristics of the studied population. Sixty-seven patients (65%) of the studied population had an HbA1c ≥ 7%. (**Table 1**). For comorbidities, there was a difference among the groups concerning the presence of HTN (p <0.05, **Table 1**). There were no statistically significant differences between the groups in age, gender, BMI, or other comorbidities. Regarding sleep parameters, there was a statistical difference for LnT90 (p < 0.05, **Table 1**), but no difference among HbA1c categories for LnAHI, and LnODI.

**Table 1:** Population characteristics.

	Overall	Controlled DM [HbA1c <7%]	Uncontrolled DM [HbA1c ≥7%]	P-value
	(N=103, 100%)	(N=36, 35%)	(N=67, 65%)	
Age*	55.5 (10.6)	54.3 (11.1)	56.1 (10.3)	0.389
**Female**	54 (52.4%)	17 (47.2%)	37 (55.2%)	0.536
BMI*	40.7 (8.11)	39.6 (7.38)	41.3 (8.47)	0.311
**HTN**	80 (77.7%)	23 (63.9%)	57 (85.1%)	<0.05*
**CAD**	17 (16.5%)	5 (13.9%)	12 (17.9%)	0.782
**AF**	8 (7.8%)	2 (5.6%)	6 (9.0%)	0.71
**HF**	8 (7.8%)	1 (2.8%)	7 (10.4%)	0.256
**Stroke**	7 (6.8%)	2 (5.6%)	5 (7.5%)	1
**CKD**	13 (12.6%)	6 (16.7%)	7 (10.4%)	0.37
LnT90*	1.57 (1.52)	1.09 (1.41)	1.82 (1.53)	<0.05*
LnAHI*	3.81 (0.549)	3.77 (0.515)	3.84 (0.569)	0.557
LnODI*	3.41 (0.809)	3.23 (0.861)	3.51 (0.768)	0.11
Comorbidities				
*Only DM*	21 (20.4%)	12 (33.3%)	9 (13.4%)	0.443
*DM + 1*	42 (40.8%)	12 (33.3%)	30 (44.8%)	
*DM + 2*	32 (31.1%)	9 (25.0%)	23 (34.3%)	
*DM + 3 or more*	8 (7.8%)	3 (8.3%)	5 (7.5%)	

*p-value <0.05.

*Summary in Mean and Standard Deviation.BMI, body mass index; HTN, systemic hypertension; CAD, coronary artery disease; AF, atrial fibrillation; HF, heart failure; CKD, chronic kidney disease; LnT90, natural log (T90+1); LnAHI, natural log (AHI+1); LnODA, natural log (ODI+1).

### 3.2 Univariate linear regression and correlations

Univariate linear regression analysis with HbA1c level as the dependent variable showed a significant positive association with female gender (ß=0.71, 95% CI= 0.12-1.3), BMI (ß=0.04, 95% CI= 0.00-0.08), and LnT90 (ß= 0.28, 95% CI= 0.09-0.47) (**Table 2**). There was no significant association between HbA1c level and LnAHI nor LnODI in the univariate analysis.

**Table 2:** Univariate analysis.

Characteristic	Beta	95% CI	p-value
Age	0.02	-0.01, 0.04	0.28
Female	0.72	0.12, 1.3	0.019*
BMI	0.04	0.00, 0.08	0.042*
LnT90	0.28	0.09, 0.47	0.005*
LnAHI	0.11	-0.45, 0.66	0.7
LnODI	0.3	-0.08, 0.67	0.12

CI, Confidence Interval.

*p-value <0.05.

### 3.3 Multivariate linear regression

The results for the multivariate analysis after adjusting for known confounders are shown in **Table 3**. Increasing levels of OSA severity measured using LnT90, and LnODI were significantly associated with increasing HbA1c after adjusting for age and gender (Model 1) with coefficient values of (ß=0.33, 95% CI= 0.14 - 0.52) and (ß=0.45, 95% CI= 0.08-0.83), respectively. The association between LnT90, LnODI and HbA1c remained significant after adjusting for BMI (Model 2), and the remaining comorbidities (Model 3). Increasing levels of the OSA indicator LnAHI on the other hand was not associated with HbA1c levels in any of the models (**Table 3**). The full models are presented in detail in the **Supplementary Material** (**Table S1**). Further, in the exploratory analysis, we found that females had higher HbA1c levels with higher levels of OSA severity when compared to males. We tested for interaction between gender and LnT90 and between gender and LnODI while controlling for age and BMI. The interaction term was significant only for the model using LnODI as the indicator, suggesting that the association between OSA severity and HbA1c was stronger in women compared to men when measured using LnODI (**Table S2**).

**Table 3:** Multivariate analysis.

	Dependent variable: HbA1c level		
	Model 1	Model 2	Model 3
LnT90	0.33*(0.14, 0.52)	0.31* (0.11, 0.50)	0.34* (0.13, 0.54)
LnAHI	0.23 (-0.32, 0.78)	0.16 (-0.39, 0.72)	0.21 (-0.39, 0.8)
LnODI	0.45* (0.08, 0.83)	0.41* (0.03, 0.79)	0.42* (0.01, 0.82)
Notes:			
Model 1	Adjusting for *age + gender*		
Model 2	Adjusting for variables in *Model 1 + BMI*		
Model 3	Adjusting for variables in *Model 2 + HTN + CAD + CKD + Stroke + HF + AF*		

*p-value <0.05.

BMI, body mass index; HTN, systemic hypertension; CAD, coronary artery disease; AF, atrial fibrillation; HF, heart failure; CKD, chronic kidney disease; LnT90, natural log (T90+1); LnAHI, natural log (AHI+1); LnODA, natural log (ODI+1).

## 4 Discussion

In the current study, we found that nocturnal hypoxemia as detected by T90 as a marker of OSA severity (18) was associated with HbA1c levels among patients with DM. Specifically, an increase in T90 and ODI levels were associated with increasing HbA1c levels even after adjusting for multiple confounders including age, gender, BMI, HTN, CAD, CKD, stroke, heart failure, and AF. However, we did not find an association between AHI levels and HbA1c.

Although several studies investigated the association between OSA and DM prevalence, few studies examined the effect of severity of OSA on glycemic control among individuals with DM (19). Thanaviratananich et al. (20) found that any degree of desaturation below 100% could adversely affect glucose metabolism. Aronsohn et al. (21) found that total AHI, AHI during REM, total ODI, and ODI during REM were all positively correlated with increasing HbA1c levels. Grimaldi et al. (22) compared the impact of OSA on HbA1c in REM vs non-REM sleep and found that total AHI and AHI during REM sleep were independently associated with increasing levels of HbA1c. They also found that increasing levels of ODI during REM was associated with increasing levels of HbA1c. Pillai et al. (23) found that increasing severity of OSA, measured using ODI, was associated with increased HbA1c levels among patients with type 2 DM. Priou et al. (24) found that increasing AHI and ODI were associated with increased HbA1c levels among untreated diabetic patients but not with treated diabetic patients. The Multi-Ethnic Study of Atherosclerosis Sleep ancillary study reported that there were significantly higher HbA1c concentrations among those in the highest quartile of hypoxemia compared with those in the lowest quartile, and there was a significant linear association between hypoxemia and HbA1c levels among patients with DM after adjustment for demographics (25). Gabryelska et al. (26) found that in patients with OSA basal oxygen saturation was independent of AHI, as a risk factor for DM.

Nocturnal hypoxemia may play an important role in developing DM. Muraki et al. (27) found in a Japanese cohort that nocturnal intermittent hypoxemia was a risk factor for the development of type 2 DM with a hazard ratio of 1.7. Bailly et al. (28) found in a cluster of OSA patients with DM that they had significant nocturnal hypoxemia with T90 reaching around 50% to 60%. Similarly, in the Jackson Heart Study, HbA1c was higher in participants with T90 of >5% (29). Kainulainen et al. (30) found among patients with severe OSA, a significant correlation between T90 and HbA1c levels. They also found that the hypoxic burden was strongly associated with the severity of daytime hypersomnolence rather than AHI (30). Further, the hypoxic burden rather than AHI strongly predicted cardiovascular mortality and all-cause mortality with a hazard ratio of 2 (31) and significantly correlated with cancer-specific mortality (32). These data support the concept that hypoxemia is more important than AHI as a predictor of adverse effects and lack of control of DM in the OSA population. On the other hand, Huang et al. (33) examined the risk of developing OSA across multiple cohorts of individuals with DM over 10 to 18 years of follow-up. They found that fasting insulin at baseline was associated with OSA. In addition, patients with DM are at increased risk for an accelerated worsening of intermittent hypoxemia. The data from the Sleep Heart Health Study showed that in the follow-up PSG after 5 years, the increase in ODI among other nocturnal hypoxemic parameters was higher among participants with DM (34).

Studies investigating the association between the prevalence of OSA among patients with DM indicate that the prevalence of OSA may be as high as 23% and that the prevalence of any form of SDB may be as high as 58% (14). Azman et al. (35) found that about 64% of their Saudi cohort of patients diagnosed with OSA had insulin resistance which was significantly correlated with increasing levels of AHI. Other studies from KSA demonstrated a high prevalence of OSA in patients with DM when screened using the STOP-BANG questionnaire where 42% of patients had moderate to high risk (36) and 53.2% were at high risk based on the Berlin questionnaire (37). Sweed et al. (38) found that 50% of a cohort of Egyptian patients with OSA had DM constituting the second most common comorbidity after HTN. Our results are following these data denoting a high prevalence of DM among a Saudi cohort with OSA.

Nocturnal hypoxemia as assessed by T90 could be linked to the development of various complications of DM and early treatment could guard against organ damage. Xue et al. (39) found that the severity of OSA was associated with higher odds of having diabetic microvascular complications. Kosseifi et al. (40) found that increasing AHI was associated with microvascular complications and retinopathy among patients with newly diagnosed OSA with well-controlled DM. Strausz et al. (11) found that patients with OSA and DM had a 1.75-fold increased risk of CKD as well as increased all-cause mortality. Accordingly, T90 could be considered a future biomarker for the development of DM complications and a good marker for follow-up of patients with DM presenting with OSA. The link between nocturnal hypoxemia and DM among OSA patients could be explained based on recurrent apneas and consequently, intermittent hypoxemia that can cause abnormality in the expression and release of cytokines from hepatocytes, myocytes, vascular endothelium, and pancreas and an inflammatory state of the adipose tissue, that contribute to insulin resistance and glucose intolerance (41, 42). On the other hand, some studies suggest a reverse causation, meaning that an increase in glycemic levels may lead to an increase in SDB. The mechanism is thought to be that poor glycemic control may lead to the deterioration of the central control of respiration and autonomic neuropathy may worsen OSA. (43–45).

We found that females had increasing levels of HbA1c compared to males with higher levels of OSA when measured using ODI as an indicator even after controlling for age and BMI levels. Compared to other studies, Priou et al. (24) found that there was no interaction between gender and OSA severity when controlling for DM treatment effects. We did not control for the treatment effect. Celen et al. (46) found in their longitudinal study that incident DM was higher in women with OSA. Alotair and BaHammam (47) found in their study comparing Saudi men and women with OSA, that women were more likely than men to be diagnosed with DM and other comorbidities including hypothyroidism, HTN, cardiac disease, and asthma. This could be explained based on older age, associated obesity, HTN (48), and more common insomnia among females rather than men (47).

The current study has some limitations. Firstly, our data were collected from patients following up at a single center sleep clinic as opposed to a community setting thus subjecting our findings to selection bias in which individuals with OSA and worse glycemic control might be more likely to be present in our study compared to individuals with OSA in a community setting. Secondly, we considered only one reading of HbA1c in our analysis rather than multiple readings at the time of OSA investigation. However, HbA1c is considered a marker of the glycemic state for an individual over several months and is a good predictor of diabetic complications (49). Thirdly, our study was a cross-sectional design, we were not able to examine temporality between exposure and effect. Finally, we did not examine the status of diabetic treatment among participants. For example, the effect of insulin and oral hypoglycemic agents, and their doses.

## 5 Conclusion

DM is commonly associated with OSA. Intermittent hypoxemia associated with obstructive events could be an important factor affecting glycemic control among patients with OSA suffering from DM irrespective of the severity of both diseases. However, this association could also be attributed to reverse causation in which glycemic control contributes to the severity of OSA.

## Data availability statement

The original contributions presented in the study are included in the article/**Supplementary Material**. Further inquiries can be directed to the corresponding author.

## Ethics statement

The study protocol was approved by the institutional review board (IRB 2020-01-136) of IAU according to the Helsinki regulations. Written informed consent for participation was not required for this study in accordance with the national legislation and the institutional requirements.

## Author contributions

All authors listed have made a substantial, direct, and intellectual contribution to the work and approved it for publication.
