# Supplementary material for: Effect of nocturnal hypoxemia on glycemic control among diabetic Saudi patients presenting with obstructive sleep apnea
Source: Front Endocrinol (Lausanne). 2023 Jan 18;13:1020617. doi: 10.3389/fendo.2022.1020617 (PMC9889975; doi:10.3389/fendo.2022.1020617)
Supplement: Supplementary file 1 [file DataSheet_1.docx]

Effect of nocturnal hypoxemia on glycemic control among diabetic Saudi patients presenting with obstructive sleep apnea – Supplementary Material

*Table S 1: Multivariate Analysis (Detailed)*

| Dependent variable: | | | |
| --- | --- | --- | --- |
| HgbA1C Level | | | |
| OLS | | | |
|  | ***Model 1*** | ***Model 2*** | ***Model 3*** |
| **Constant** | 6.72* (5.22, 8.22) | 6.07* (3.86, 8.28) | 5.94* (3.66, 8.21) |
| **LnT90** | 0.33* (0.14, 0.52) | 0.31* (0.11, 0.50) | 0.34* (0.13, 0.54) |
| **Age** | 0.003 (-0.02, 0.03) | 0.01 (-0.02, 0.03) | -0.001 (-0.03, 0.03) |
| **Female** | 0.88* (0.30, 1.45) | 0.78* (0.16, 1.40) | 0.79* (0.11, 1.46) |
| **BMI** |  | 0.02 (-0.02, 0.05) | 0.01 (-0.02, 0.05) |
| **HTN** |  |  | 0.55 (-0.19, 1.30) |
| **CAD** |  |  | 0.31 (-0.52, 1.15) |
| **CKD** |  |  | -0.30 (-1.21, 0.60) |
| **Stroke** |  |  | -0.28 (-1.45, 0.90) |
| **HF** |  |  | 0.39 (-0.72, 1.49) |
| **AF** |  |  | 0.27 (-0.84, 1.38) |
|  | | | |
| Observations | 103 | 103 | 103 |
| R2 | 0.16 | 0.16 | 0.2 |
| Adjusted R2 | 0.13 | 0.13 | 0.12 |
| Residual Std. Error | 1.45 (df = 99) | 1.45 (df = 98) | 1.46 (df = 92) |
| F Statistic | 6.10** (df = 3; 99) | 4.71** (df = 4; 98) | 2.35* (df = 10; 92) |

*Note: p<0.05, LnT90: Natural log (T90+1)

| *Table S 1 (cont.…): Multivariate Analysis (Detailed)* | | | |
| --- | --- | --- | --- |
| Dependent variable: | | | |
| HgbA1C Level | | | |
| OLS | | | |
|  | ***Model 1*** | ***Model 2*** | ***Model 3*** |
| **Constant** | 5.98* (3.37, 8.58) | 5.05* (2.14, 7.96) | 4.75* (1.68, 7.82) |
| **LnAHI** | 0.23 (-0.32, 0.78) | 0.16 (-0.39, 0.72) | 0.21 (-0.39, 0.80) |
| **Age** | 0.01 (-0.02, 0.04) | 0.01 (-0.01, 0.04) | 0.01 (-0.02, 0.04) |
| **Female** | 0.74* (0.13, 1.34) | 0.57 (-0.08, 1.22) | 0.51 (-0.18, 1.21) |
| **BMI** |  | 0.03 (-0.01, 0.07) | 0.03 (-0.01, 0.07) |
| **HTN** |  |  | 0.51 (-0.28, 1.30) |
| **CAD** |  |  | 0.21 (-0.66, 1.09) |
| **CKD** |  |  | -0.21 (-1.15, 0.74) |
| **Stroke** |  |  | -0.47 (-1.72, 0.79) |
| **HF** |  |  | 0.33 (-0.86, 1.53) |
| **AF** |  |  | -0.004 (-1.16, 1.16) |
|  | | | |
| Observations | 103 | 103 | 103 |
| R2 | 0.07 | 0.08 | 0.12 |
| Adjusted R2 | 0.04 | 0.05 | 0.02 |
| Residual Std. Error | 1.52 (df = 99) | 1.51 (df = 98) | 1.54 (df = 92) |
| F Statistic | 2.35 (df = 3; 99) | 2.25 (df = 4; 98) | 1.21 (df = 10; 92) |

*Note: p<0.05, LnAHI: Natural log (AHI+1)

| *Table S 1 (cont.…): Multivariate Analysis (Detailed)* | | | |  |
| --- | --- | --- | --- | --- |
| Dependent variable: | | | |  |
| HbA1C Level | | | |  |
| OLS | | | |  |
|  | ***Model 1*** | ***Model 2*** | ***Model 3*** | |
| **Constant** | 5.28* (3.28, 7.29) | 4.57* (2.15, 7.00) | 4.40* (1.87, 6.93) | |
| **LnODI** | 0.45* (0.08, 0.83) | 0.41* (0.03, 0.79) | 0.42* (0.01, 0.82) | |
| **Age** | 0.01 (-0.02, 0.04) | 0.01 (-0.02, 0.04) | 0.01 (-0.02, 0.04) | |
| **Female** | 0.90* (0.29, 1.50) | 0.77* (0.11, 1.42) | 0.70 (-0.01, 1.40) | |
| **BMI** |  | 0.02 (-0.02, 0.06) | 0.02 (-0.02, 0.06) | |
| **HTN** |  |  | 0.39 (-0.39, 1.17) | |
| **CAD** |  |  | 0.16 (-0.70, 1.02) | |
| **CKD** |  |  | -0.22 (-1.15, 0.71) | |
| **Stroke** |  |  | -0.57 (-1.79, 0.66) | |
| **HF** |  |  | 0.43 (-0.72, 1.58) | |
| **AF** |  |  | 0.04 (-1.10, 1.17) | |
|  | | | | |
| Observations | 103 | 103 | 103 | |
| R2 | 0.11 | 0.12 | 0.15 | |
| Adjusted R2 | 0.08 | 0.08 | 0.06 | |
| Residual Std. Error | 1.48 (df = 99) | 1.48 (df = 98) | 1.51 (df = 92) | |
| F Statistic | 4.13** (df = 3; 99) | 3.36* (df = 4; 98) | 1.62 (df = 10; 92) | |

*Note: p<0.05, LnODI: Natural log (ODI+1)

*Table S 2: Multivariate Analysis with Interaction Term between Sex and OSA*

| *Dependent variable:*  HbA1C Level | | | |
| --- | --- | --- | --- |
| *OLS* | | | |
|  | (1) |  | (2) |
|  | | | |
| **Constant** | 6.46* (4.20, 8.72) | **Constant** | 6.13* (3.28, 8.97) |
| **Age** | 0.003 (-0.02, 0.03) | **Age** | 0.01 (-0.02, 0.04) |
| **BMI** | 0.01 (-0.02, 0.05) | **BMI** | 0.02 (-0.02, 0.06) |
| **Female** | 0.36 (-0.48, 1.21) | **Female** | -1.75 (-4.32, 0.83) |
| **LnT90** | 0.18 (-0.09, 0.44) | **LnODI** | 0.05 (-0.47, 0.57) |
| **Female*LnT90** | 0.27 (-0.10, 0.65) | **Female*LnODI** | **0.74* (0.01, 1.48)** |
|  | | | |
| Observations | 103 | Observations | 103 |
| R^2^ | 0.18 | R^2^ | 0.15 |
| Adjusted R^2^ | 0.14 | Adjusted R^2^ | 0.11 |
| Residual Std. Error (df = 98) | 1.44 | Residual Std. Error (df = 98) | 1.46 |
| F Statistic (df = 5; 98) | 4.22^*^ | F Statistic (df = 5; 98) | 3.55^*^ |
|  | | | |
| *Note:* *p<0.05, LnT90: Natural log (T90+1), LnODI: Natural log (ODI+1) | | | |
